# Red Blood Cell Morphodynamics: A New Potential Marker in High-Risk Patients

**DOI:** 10.3389/fphys.2020.603633

**Published:** 2021-01-13

**Authors:** Benedetta Porro, Edoardo Conte, Anna Zaninoni, Paola Bianchi, Fabrizio Veglia, Simone Barbieri, Susanna Fiorelli, Sonia Eligini, Alessandro Di Minno, Saima Mushtaq, Elena Tremoli, Viviana Cavalca, Daniele Andreini

**Affiliations:** ^1^Centro Cardiologico Monzino, Istituto di Ricovero e Cura a Carattere Scientifico (IRCCS), Milan, Italy; ^2^Fondazione IRCCS Ca’ Granda Ospedale Maggiore Policlinico Milano, Unità Operativa Complessa (UOC) Ematologia, Unità Operativa Semplice (UOS) Fisiopatologia delle Anemie, Milan, Italy; ^3^Department of Clinical Sciences and Community Health, Cardiovascular Section, University of Milan, Milan, Italy

**Keywords:** red blood cell morphodynamics, non-obstructive coronary artery disease, high-risk plaque, coronary computed tomography angiography, risk chart

## Abstract

In the last years, a substantial contribution of red blood cells (RBCs) in cardiovascular homeostasis has been evidenced, as these cells are able to regulate cardiovascular function by the export of adenosine triphosphate and nitric oxide as well as to maintain redox balance through a well-developed antioxidant system. Recently a link between high-risk plaque (HRP) features and myocardial ischemia, in the absence of severe lumen stenosis, has been evidenced. Nonobstructive coronary artery disease (nonob CAD) has been associated in fact with a greater 1-year risk of myocardial infarction and all-cause mortality compared with no apparent CAD. This new evidence increases interest in searching new triggers to identify these high-risk patients, in the absence/or on top of traditional hazard markers. In this study, we investigated the existence of any association between RBC morphodynamics and HRP features in individuals with different grades of coronary stenosis detected by coronary computed tomography angiography (CCTA). Ninety-one consecutive individuals who underwent CCTA [33 no CAD; 26 nonobstructive (nonob), and 32 obstructive (ob) CAD] were enrolled. RBC morphodynamic features, i.e., RBC aggregability and deformability, were analyzed by means of Laser Assisted Optical Rotation Cell Analyzer (LoRRca MaxSis). The putative global RBC morphodynamic (RMD) score and the related risk chart, associating the extent of HRP (e.g., the non-calcified plaque volume) with both the RMD score and the max % stenosis were computed. In nonob CAD group only positive correlations between RBC rigidity, osmotic fragility or aggregability and HRP features (plaque necrotic core, fibro-fatty and fibro-fatty plus necrotic core plaque volumes) were highlighted. Interestingly, in this patient cohort three of these RBC morphodynamic features result to be independent predictors of the presence of non-calcified plaque volume in this patients group. The risk chart created shows that only in nonob CAD plaque vulnerability increases according to the score quartile. Findings of this work, by evidencing the association between erythrocyte morphodynamic characteristics assessed by LoRRca and plaque instability in a high-risk cohort of nonob CAD, suggest the use of these blood cell features in the identification of high-risk patients, in the absence of severe coronary stenosis.

## Introduction

Coronary artery disease (CAD) is one of the major causes of morbidity and mortality in the western countries (Montalescot et al., 2013). Specific anatomic plaque features have been established as fundamental to the process leading to acute coronary thrombosis and, among them, plaque burden, thin-cap fibroatheroma, positive arterial remodeling, necrotic cores, spotty calcifications, and macrophage infiltration play a central role (Virmani et al., 2006).

Although the diagnosis of obstructive (ob) CAD is the milestone for risk stratification in cardiac disease, non-obstructive (nonob) CAD is a relatively common feature, occurring in 10–25% of patients undergoing coronary angiography (Bugiardini and Bairey Merz, 2005), and its presence has been defined as “insignificant” or “no significant CAD” in the medical literature so far (Hung and Cherng, 2003; Patel et al., 2006). Nevertheless, a high number of atheromatous plaques that are not flow limiting are responsible for acute coronary syndromes (ACS; Finn et al., 2010).

The need for improved methods and new markers beyond stenosis for high-risk plaque (HRP) identification follows from these premises.

In the last years, coronary computed tomography angiography (CCTA) has emerged as a non-invasive method for accurate detection and/or exclusion of the presence of CAD (Al-Mallah et al., 2015). Specifically, a high extent of literature evidenced the ability of CCTA to identify not only ob CAD but also early atherosclerotic lesions (Min et al., 2011; Andreini et al., 2012). As before the “CCTA era” patients with nonob CAD and without signs of inducible ischemia were included in the same group of those without evident disease, now with the aid of this imaging technique, we are able, in this patient group, to discriminate between individuals with low-risk plaque morphology and subjects in whom plaque characteristics are associated with an increased risk of future events (Libby, 2013; Conte et al., 2017).

These methodological improvements enhanced a new interest in the evaluation of atherosclerosis determinants, from lumen stenosis to myocardial ischemia. In this context, a new and emerging factor is red blood cell (RBC), not only the transporter of oxygen to tissues but also a cell able to modulate blood flow behavior. Several factors related to RBCs are associated with CAD including erythrocyte sedimentation rate, hemoglobin levels, hematocrit (Hct), and red blood cell distribution width (RDW; Danesh et al., 2000). In particular, it has been demonstrated that men who had the erythrocyte sedimentation rate in the upper quintile had more than twice the risk of coronary heart disease (CHD) death (Gillum et al., 1995). As regards hemoglobin, an observational study conducted on 2,059 patients undergoing coronary artery bypass surgery revealed that individuals with a preoperative hemoglobin concentration of 100 g/L or less had a five-fold higher in-hospital mortality rate after surgery than those with a higher hemoglobin concentration (Zindrou et al., 2002). Opposite results were published on the correlation between Hct and the risk of CHD, with high Hct level associated with an increased risk of myocardial infarction, coronary insufficiency, or CHD death (Sorlie et al., 1981). Recently, the role of RDW in identifying mortality and cardiovascular risk among patients with CAD has been highlighted, being high RDW levels associated with increased risk of mortality and cardiovascular disease (CVD) events in patients with established CAD (Su et al., 2014). However, scarce knowledge is available nowadays regarding their functional profile in relation to morphodynamic features.

An increase in RBC aggregation has been described in patients with acute myocardial infarction (Lakshmi et al., 2011) and was associated with different cardiovascular risk factors, i.e., age (Vaya et al., 2013), obesity (Wiewiora et al., 2007), or diabetes mellitus (Martinez et al., 1998).

A reduced RBC deformability was reported in different vessel types (Baskurt and Meiselman, 2003; Keymel et al., 2011) and linked with pathological states related to microcirculatory disorders such as CAD (Pytel et al., 2013), hypertension (Odashiro et al., 2015), hypercholesterolemia (Kohno et al., 1997), and diabetes mellitus (Jain and Lim, 2000).

However, no data are available about the existence of a relation between RBC rheological properties and high-risk atherosclerotic plaque features.

In this study, we investigated the morphodynamic features of erythrocytes of individuals with different grades of coronary stenosis by means of Laser Assisted Optical Rotational Cell Analyzer (LoRRca MaxSis) in order to find any association between RBC features and plaque characteristics.

## Materials and Methods

### Study Population and Blood Collection

In this study, we prospectively enrolled 91 consecutive patients who underwent CCTA between March 2016 and February 2018 for suspected but unknown stable CAD. In all patients, blood sample was obtained before CCTA and collected into EDTA tubes. Based on CCTA evaluation, patients were defined as having no apparent CAD in the absence of any plaque in the coronary tree (0% stenosis and no luminal irregularities, namely no CAD). Nonobstructive disease was defined as the presence of limited atherosclerotic disease demonstrated by a stenosis <50% (1–49%, named nonob CAD). When the atherosclerotic disease was associated with a stenosis ≥50%, patients were classified as ob CAD.

All patients were further evaluated for the presence of traditional cardiovascular risk factors such as diabetes mellitus (fasting glucose level of 126 mg/dl or higher and/or the need for insulin or oral hypoglycemic agents), hypercholesterolemia (total cholesterol level >200 mg/dl, or treatment with lipid-lowering drugs), hypertension, smoking attitude, and family history of CAD.

This observational study was carried out in accordance with the Declaration of Helsinki and approved by the local ethics research committee of Centro Cardiologico Monzino. Written informed consent to participate was obtained from all subjects.

### Coronary Computed Tomography Angiography Scan Protocol, Images Reconstruction, and Analysis

Before CT scan, patients were treated with intravenous beta-blocker (Metoprolol up to 20 mg) to optimize heart rate and with a standard dose of sublingual nitrates. CCTAs were performed using a last generation 256-CT scanner (Revolution CT GE Healthcare, Milwaukee, WI, USA) with prospective ECG-triggering. A BMI-adapted scanning protocol was used: BMI < 20 Kg/m^2^, tube voltage and tube current of 100 kVp and 500 mA, respectively; 20 ≤ BMI < 25 Kg/m^2^, tube voltage and tube current of 100 kVp and 550 mA, respectively; 25 ≤ BMI < 30 Kg/m^2^, tube voltage and tube current of 100 kVp and 600 mA, respectively; 30 ≤ BMI < 35 Kg/m^2^, tube voltage and tube current of 120 kVp and 650 mA, respectively. Patients received a 50-ml (for BMI ≤ 25 Kg/m^2^) or 60-ml (for BMI > 25 Kg/m^2^) bolus of contrast medium (Iomeron 400 mg/ml, Bracco, Milan, Italy) through an antecubital vein at an infusion rate of 5 ml/s, followed by 50 ml of saline solution. The imaging was performed using bolus tracking technique.

Image CCTA datasets were evaluated using vessel analysis software (CardioQ3 Package - GE Healthcare).

Coronary plaques were defined as structures of at least 1 mm^2^ area adjacent to coronary lumen, clearly distinguishable from the vessel lumen and surrounded by pericardial tissue; tissue with signal intensity below 40 HU was considered a pericardial fat and excluded from analysis. Normal coronary arteries were defined when no atherosclerotic plaque (including focal and eccentric calcified plaques) could be detected in any segment within the coronary artery wall or lumen.

Advanced coronary atherosclerosis evaluation were performed as follows: arterial remodeling index assessed using vessel area = lesion plaque area/reference area, plaque burden = (lesion plaque area-lesion lumen area)/lesion plaque area, napkin ring sign defined as the presence of a semi-circular thin enhancement around the plaque along the outer contour of the vessel, and small spotty calcifications as any discrete calcification ≤3 mm in length and occupying ≤90° arc when viewed in short axis, low-attenuation plaque defined as the presence of any plaque voxel <30 HU 11. Total plaque volume was evaluated and reported in mm^3^ as previously described (Conte et al., 2020b). Non-calcified fibro-fatty plaque volume was expressed as the amount of plaque <150 HU, reported in mm^3^.

### Measurement of Morphodynamic RBC Characteristics

The analysis of RBC morphodynamic features was performed by means of Laser Assisted Optical Rotational Red Cell Analyzer (LoRRca MaxSis, Mechatronics, Hoorn, The Netherlands) according to the manufacturer’s instructions and detailed below.

Osmotic gradient dependent RBC deformability: 250 μl of whole blood was suspended in 5 ml of polyvinylpyrrolidone buffer (Mechatronics, Hoorn, The Netherlands) and used for the analysis. The osmotic gradient curve generated by the instrument shows the variation in deformability as a continuous function of the osmolality of the solution in which RBCs are dissolved. The following parameters were evaluated: the elongation index (EI)max, corresponding to the maximal deformability or elongation obtained near the isotonic osmolality and is an expression of the membrane surface; the EImin, corresponding to the osmolality at which the deformability reaches its minimum and represents the 50% of the RBCs hemolysis in conventional osmotic fragility assays, reflecting mean cellular surface-to-volume ratio; the osmolality (O)EImax or Omin, corresponding to the osmolality value at which the deformability reaches its EImax or EImin; the area under the curve (AUC, reported in the text as Area), defined in the provided software as the AUC beginning from a starting point in the hypo-osmolar region and an ending point in the hyper-osmolar region (instrument settings 500 mOsm/kg; Baskurt and Meiselman, 2004; Baskurt et al., 2009).

Red blood cells aggregation and disaggregation: 1 ml of oxygenated blood was placed into a preheated (37°C) Couette system consisting of two cylinders. A photo diode, integrated in the fixed inner cylinder, detects the intensity of the backscattered light during the RBC aggregation and disaggregation processes. A syllectogram curve was generated at the end of the assay and the following parameters were automatically calculated: the amplitude (Amp), showing the total extent of aggregation; the aggregation index (AI), calculated as integral of the total syllectogram curve; and the aggregation halftime (t1/2), reflecting the kinetics of RBC aggregation.

### Statistical Analysis

Continuous variables were presented as mean ± SD or as median with interquartile range [IQR: 25°–75°], if more appropriate. Continuous variables normally distributed were compared using the Student’s *t*-test for independent samples. When the variable distribution was not normal, Mann-Whitney U tests for independent samples were used. Variables with positively skewed distributions were log-transformed before analysis. The proportion of the categorical variables was compared using a *χ*^2^ analysis or Fisher exact test, as appropriate. A value of *p* < 0.05 was considered statistically significant.

Logistic regression analysis was used in order to evaluate the relationship between biological variables and CCTA advanced coronary atherosclerosis characteristics (i.e., plaque volume and HRP features).

For every biological variable associated with CCTA finding with a *p* < 0.10 at logistic regression analysis, a receiver operating characteristic (ROC) curve and a correlation analysis were performed.

The association between RBC morphodynamics and HRP features was assessed by multivariable general linear models, after stratifying the population into the three mentioned groups. The hypothesis that RBC morphodynamics is predictive of a HRP in nonob CAD patients but not in ob ones was tested by computing the appropriate interaction terms. The different RBC morphodynamics variables were tested individually and by multiple regression. Statistical analysis and graphics were produced with MedCalc (version 11.6.1.0, Med-Calc Software; 1993–2011) and by SAS v. 9.4 statistical package (SAS Inc. Cary NC, USA).

#### Global RBC Morphodynamic (RMD) Score

In order to summarize the potential association of the different RBC morphodynamic variables with HRP in nonob CAD patients, we created a RMD score with the following procedure: first, we ran a multiple regression with non-calcified plaque volume as dependent variable and the RBC morphodynamic variables as predictors; then, the score was computed as the sum of the variables that were independent predictors of non-calcified plaque volume in the multivariable analysis, each weighted by the beta coefficient. The strength of the associations of the individual variables and of the score with HRP was quantified by the partial R-square.

## Results

### Population Features

A total of 91 patients were consecutively enrolled. Male prevalence was 68.1% (62 out of 91) and mean age was 61.07 ± 10.9 years. The presence of plaque was excluded in 33 patients (36.3%), while 26 patients had nonob CAD (28.6%) and 32 patients had at least one obstructive coronary stenosis (35.2%).

All demographic and clinical characteristics of the study population were reported in Table 1.

**Table 1:** Demographic and clinical characteristics of the study population.

Variables	All patients *N* = 91	no CAD *N* = 33	nonob CAD *N* = 26	ob CAD *N* = 32	*p*
**Demographic and clinical characteristics**					
Age, years	61.07 ± 10.9	57.1 ± 11.5	60.68 ± 10	65.48 ± 9.7^*^	0.007
Male, *n* (%)	62 (68.1)	18 (54.5)	20 (76.9)	24 (75)	0.07
BMI, kg/m^2^	25.42 ± 3.8	25.5 ± 4.5	25.01 ± 3.6	25.66 ± 3.3	0.80
WBC, 10^3^/μl	7.41 ± 2.2	7.78 ± 2.5	7.3 ± 1.9	7.11 ± 2.2	0.46
Platelet, 10^3^/μl	221.34 ± 61.8	242.76 ± 70	213.69 ± 55.7	205.47 ± 52^*^	0.04
MPV, fl	10.36 ± 1.4	10.55 ± 0.8	10.23 ± 0.9	10.27 ± 2	0.60
RBC, 10^6^/μl	4.75 ± 0.6	4.69 ± 0.5	4.83 ± 0.5	4.75 ± 0.8	0.72
Hb, g/dl	14.3 [13.2–15.2]	13.7 [13–14.6]	14.5 [13.9–15.6]	14.4 [13.4–15.4]	0.05
Hct, %	41.48 ± 4.6	40.4 ± 3.2	42.33 ± 3.7	41.91 ± 6.2	0.22
MCV, fl	87.56 ± 4.5	86.39 ± 5.5	88 ± 4.5	88.41 ± 3.1	0.17
MCH, pg	30.22 ± 1.8	29.49 ± 2.1	30.5 ± 1.9^*^	30.75 ± 1.2^*^	0.01
MCHC, %	34.51 ± 0.9	34.13 ± 1	34.66 ± 0.9^*^	34.78 ± 0.8^*^	0.01
RDW-CV, %	13.17 ± 0.9	13.22 ± 0.9	13.12 ± 0.7	13.15 ± 0.9	0.90
RDW-SD, fl	41.32 ± 2.8	40.84 ± 2.1	41.58 ± 3	41.59 ± 3.2	0.47
Total cholesterol, mg/dl	197.8 ± 39.5	196.3 ± 35.2	187.9 ± 46.8	206.8 ± 36.1	0.22
LDL cholesterol, mg/dl	118.5 ± 35.1	116.9 ± 29.1	106.7 ± 42.5	129.3 ± 31.4	0.05
HDL cholesterol, mg/dl	57.4 ± 15.1	60.2 ± 14.8	57.2 ± 18.6	54.8 ± 12.3	0.36
Triglycerides, mg/dl	104.77 ± 49.1	97.61 ± 47.6	103.19 ± 50.8	113.44 ± 49.5	0.43
Basal glucose, mg/dl	99.5 ± 16.7	103.3 ± 19.7	93 ± 10.4	100.8 ± 16.2	0.05
Hypertension, *n* (%)	43 (47.3)	14 (42.4)	12 (46.2)	17 (53.1)	0.39
Family history of CVD, *n* (%)	36 (39.6)	10 (30.3)	15 (57.7)	11(34.4)	0.65
Dyslipidaemia, *n* (%)	37 (40.6)	13 (39.4)	14 (53.8)	10 (31.2)	0.51
Diabetes, *n* (%)	5 (5.5)	2 (6.1)	1 (3.8)	2 (6.2)	0.98
Active smokers, *n* (%)	20 (21.9)	5 (15.2)	9 (34.6)	6 (18.7)	0.17
Past smokers, *n* (%)	16 (17.6)	7 (21.2)	2 (7.7)	7 (21.8)	0.28
**Pharmacological treatments**					
β-blockers, *n* (%)	55 (60.4)	16 (48.5)	19 (71.1)	20 (62.5)	0.24
ACE-inhibitors, *n* (%)	18 (19.8)	6 (18.2)	6 (23.1)	6 (18.7)	0.95
Angiotensin receptor blockers, *n* (%)	16 (17.6)	4 (12.1)	5 (19.2)	7 (21.8)	0.30
Diuretics, *n* (%)	13 (16.3)	4 (12.1)	1 (3.8)	8 (25)	0.14
Aspirin, *n* (%)	20 (21.9)	8 (24.4)	4 (15.4)	8 (25)	0.95
Statins, *n* (%)	30 (32.9)	7 (21.1)	13 (50)	10 (31.2)	0.38

BMI, body mass index; CVD, cardiovascular disease; Hb, hemoglobin; Hct, hematocrit; HDL, high-density lipoprotein; LDL, low-density lipoprotein; MCH, mean corpuscular hemoglobin; MCHC, mean corpuscular hemoglobin concentration; MCV, mean corpuscular volume; MPV, mean platelet volume; RBC, red blood cell; RDW-CV, red blood cell distribution width-coefficient of variation; RDW-SD, red blood cell distribution width-standard deviation; WBC, white blood cell.

* *p* < 0.05 vs. no CAD;

† *p* < 0.05 vs. nonob CAD.

Patients with ob CAD were older compared to individuals without CAD. This difference is reflected in a low number of platelets in ob CAD individuals. Interestingly, both ob and nonob CAD patients showed higher mean corpuscular hemoglobin (MCH) and mean corpuscular hemoglobin concentration (MCHC) values compared to no CAD individuals. Prevalence of traditional risk factors and medical therapy at the time of cardiac CCTA were similar throughout the entire enrolled population.

### CCTA Characteristics

In Table 2, the main features of atherosclerotic lesions detected by CCTA have been reported.

**Table 2:** CCTA characteristics of the study population.

Parameters	All patients *N* = 91	no CAD *N* = 33	nonob CAD *N* = 26	ob CAD *N* = 32	*p*
Total plaque volume, mm^3^	88.5 ± 128.2	-	99.6 ± 126.7^*^	170.7 ± 139.1^*^^,^^†^	<0.001
Non-calcified plaque volume, mm^3^	25.2 ± 40.7	-	25.9 ± 35.6^*^	50.7 ± 49.3^*^^,^^†^	<0.001
High risk plaque features >2, *n* (%)	26 (28.6)	0	6 (23.1)	20 (62.5)^*^^,^^†^	<0.001
Non-calcified plaque volume HQ, *n* (%)	23 (25.3)	0	6 (23.1)	17 (53.1)^*^^,^^†^	<0.001
Total plaque volume HQ, *n* (%)	23 (25.3)	0	5 (19.2)	18 (56.3)^*^^,^^†^	<0.001

CAD, coronary artery disease; CCTA, coronary computed tomography angiography; HQ, high quartile.

* *p* < 0.05 vs. no CAD;

† *p* < 0.05 vs. nonob CAD.

Total coronary plaque volume was 88.5 ± 128.2 mm^3^ in the whole cohort of patients enrolled in the study and resulted to be significantly higher in patients with ob CAD when compared to those with nonob disease (*p* < 0.0001). Similarly, non-calcified plaque volume resulted to be significantly higher in patients with ob CAD than in nonob CAD ones. In 26 of 91 patients (28.6%) more than two HRP features were identified; as expected, an high prevalence of HRP features was recorded among patients with ob CAD [6 (23.1%) vs. 20 (62.5%), value of *p* < 0.0001 for nonob vs. ob CAD patients].

### RBC Morphodynamics

In our study population, the analysis of RBC morphodynamic characteristics in relation to the degree of stenosis did not evidence any difference. The only parameter altered in ob CAD patients compared to no CAD is the Area (value of *p* = 0.024, Table 3), evidencing a reduced ability of erythrocytes to modify their shape in response to an osmotic gradient curve in patients with a severe stenosis.

**Table 3:** RBC morphodynamic parameters in the study population.

Parameters	All patients *N* = 91	no CAD *N* = 33	nonob CAD *N* = 26	ob CAD *N* = 32	*p*
***Deformability***					
EImax	0.63 ± 0.02	0.62 ± 0.02	0.63 ± 0.01	0.63 ± 0.02	0.54
EImin	0.12 ± 0.01	0.12 ± 0.01	0.12 ± 0.01	0.12 ± 0.01	0.96
O EImax, mOsm/kg	290.85 ± 19.5	290.96 ± 18.4	285.95 ± 17.1	294.84 ± 22.1	0.31
Omin, mOsm/kg	134.81 ± 8.4	134.23 ± 7.8	133.81 ± 8.5	136.24 ± 9.1	0.57
Area	149.93 ± 5.2	152.02 ± 5.3	149.45 ± 4.9	148.14 ± 4.7^*^	0.02
***Aggregation***					
AI, %	64.76 ± 10.4	66.69 ± 8.7	61.81 ± 11.2	65.54 ± 10.8	0.24
Amp, au	37.09 ± 9.5	37.02 ± 11.6	39.79 ± 5.2	34.94 ± 10	0.19
t_1/2_, sec	1.8 [1.5–2.5]	1.8 [1.3–2.3]	2.2 [1.6–2.5]	1.8 [1.3–2.4]	0.17
RMD score	−47.2 [−47.6, −47.0]	−47.1 [−47.6, −46.9]	−47.2 [−47.5, −47.0]	−47.3 [−48.1, −46.7]	0.75

AI, aggregation index; Amp, amplitude of the aggregate; CAD, coronary artery disease; EImax, maximal elongation index; EImin, minimum elongation index; O EImax, osmolality corresponding to maximal elongation index; Omin osmolality corresponding to minimum elongation index; RMD, RBC morphodynamic; t1/2, aggregation halftime.

* *p* < 0.05 vs. no CAD.

### RBC Morphodynamics and Atherosclerotic Plaque Features at CCTA

While no statistically significant association between RBC morphodynamic characteristics assessed by LoRRca and HRP features was found in patients with severe stenosis, in nonob CAD group, several relationships were evidenced.

**Figure 1:**
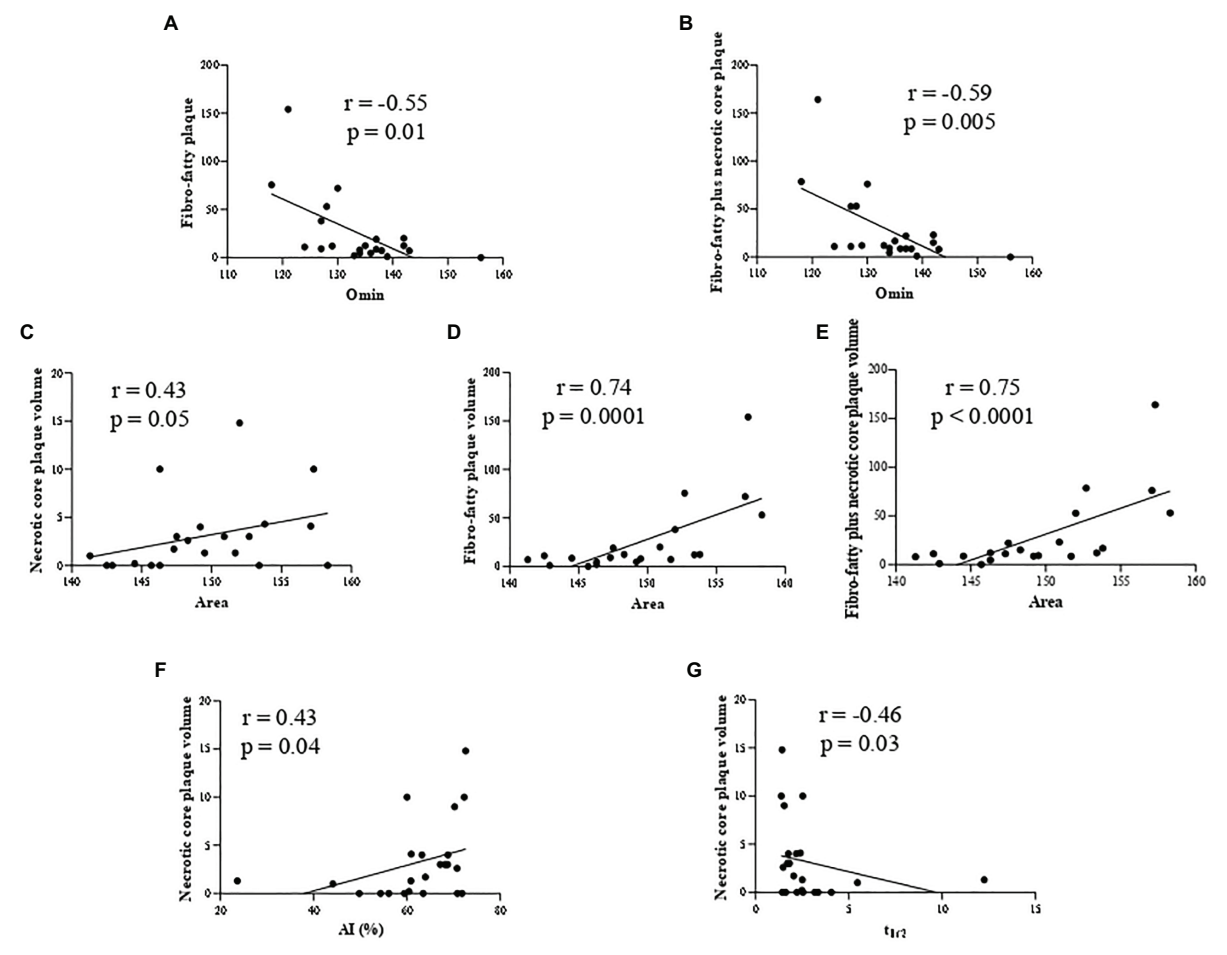
Correlation analysis in nonob CAD patients revealing the association between: RBC osmotic fragility (evaluated using the Omin value from the osmoscan curve) and two HRP features (fibro-fatty **(A)** and fibro-fatty plus necrotic core plaque **(B)** volumes) and between RBC rigidity (assessed by a reduction in the Area of the osmoscan graph) and necrotic core **(C)**, fibro-fatty **(D)** and fibro-fatty plus necrotic core **(E)** plaque volumes. RBC from nonob CAD with a high necrotic core plaque volume displayed an increased kinetics of aggregation [displayed by AI **(F)** and t_1/2_ **(G)**]. AI, aggregation index; Area, area under the elongation index-related osmolality curve; CAD, coronary artery disease; HRP, high-risk plaque; nonob CAD, non-obstructive coronary artery disease; Omin, minimal osmolality; RBC, red blood cell; t_1/2_, aggregation half time.

In particular, we highlighted a positive correlation between RBC osmotic fragility (evaluated using the Omin value from the osmoscan curve) and two HRP features: fibro-fatty (Figure 1A) and fibro-fatty plus necrotic core plaque (Figure 1B) volumes. In parallel with increased fragility, we found correlations between RBC rigidity (assessed by a reduction in the Area of the osmoscan graph), defined by the inability of cell to change its shape under various osmotic conditions, and necrotic core (Figure 1C), fibro-fatty (Figure 1D) and fibro-fatty plus necrotic core (Figure 1E) plaque volumes.

In addition, RBC from nonob CAD patients with a high necrotic core plaque volume displayed an increased kinetics of aggregation (Figure 1F,G).

In accordance to these findings, we evidenced only in nonob CAD group the existence of an interaction among all these morphodynamic RBC characteristics and fibro-fatty plus necrotic core plaque volume (value of *p* = 0.005).

**Figure 2:**
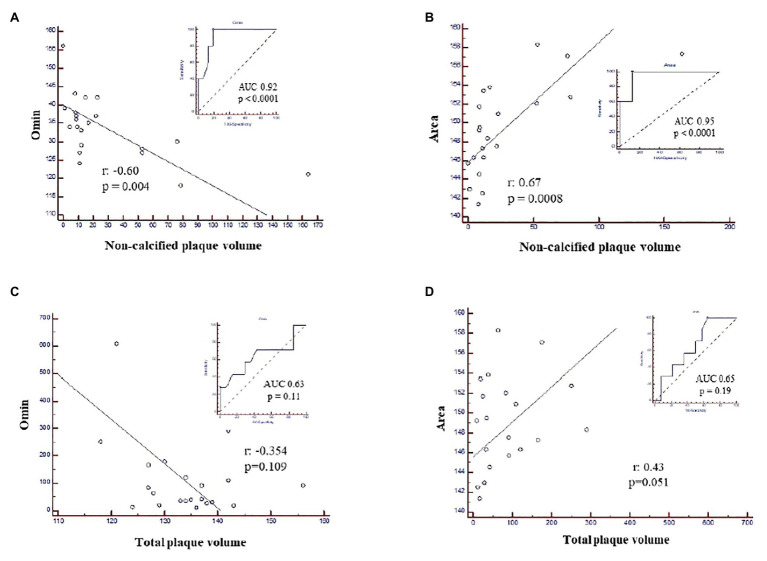
ROC curves and correlation analysis in nonob CAD patients showing the association between non-calcified plaque volume and **(A)** Omin, corresponding to the degree of RBC resistance to lysis; **(B)** the Area and the absence of any correlation between total plaque volume and Omin **(C)** and the Area **(D)**. Area, area under the elongation index-related osmolality curve; CAD, coronary artery disease; nonob CAD, non-obstructive coronary artery disease; Omin, minimal osmolality; RBC, red blood cell; ROC, Receiver Operating Characteristic.

In this patient group, the non-calcified plaque volume resulted to be inversely associated with the degree of RBC resistance to lysis (Omin; Figure 2A) and positively correlated with the Area (Figure 2B), while no association was found with the total plaque volume (Figures 2C,D), an index of the extent of atherosclerosis.

Of interest, neither Omin nor Area or RBC aggregation parameters resulted to be significantly correlated to HRP features among patients with ob CAD (Supplementary Figures S1, S2).

**Figure 3:**
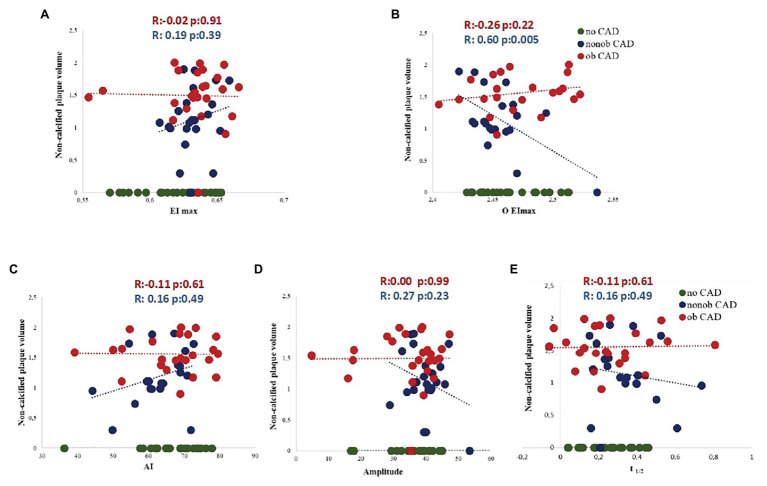
Association between five RBC morphodynamic characteristics (AI, **A**; Amp, **B**; t_1/2_, **C**; EImax, **D**; O EImax, **E**) and non-calcified plaque volume in nonob CAD (red dots), ob CAD (green dots) and no CAD (blue dots) patients. AI, aggregation index; Amp, amplitude of RBC aggregation; CAD, coronary artery disease; EImax, maximal elongation index; nonob CAD, non-obstructive coronary artery disease; O EImax, osmolality for EImax; ob CAD, obstructive coronary artery disease; RBC, red blood cell; t_1/2_, aggregation half time.

To corroborate the existence of a relationship between RBC morphodynamic characteristics and HRP features specifically in nonob CAD population, in this patient group, we found an apparent linear relation between five RBC morphodynamic features and non-calcified plaque volume. Although the correlation was significant only for O EImax, the pattern was similar for all features, with a concomitant totally flat relation in patients with severe stenosis (ob CAD; Figures 3A–E).

Most importantly, in a multivariable analysis, three morphodynamic RBC variables were independent predictors of non-calcified plaque volume in nonob CAD patients, i.e., EImax, O EImax and t_1/2_ (Table 4). These variables were included in the putative global RMD score, which was computed using the following formula:

$$\begin{array}{c} \mathrm{RMD}\;\mathrm{score}=22.6\times \log\left(\mathrm{EImax}\right)+17.2\times \log\left(O\;\mathrm{EImax}\right)+\\ 1.6\times \log\left(t1/2\right) \end{array}$$

**Table 4:** Morphodynamic RBC variables that are independent predictors of non-calcified plaque volume in nonob CAD patients.

Variable (log-transformed)	Beta coefficient	SE	*p*
EImax	22.6	7.7	0.011
O EImax	17.2	2.8	<0.0001
t_1/2_	1.6	0.5	0.004

EI max, maximal elongation index; O EImax, osmolality corresponding to maximal elongation index; SE, standard error; t1/2, aggregation halftime.

**Figure 4:**
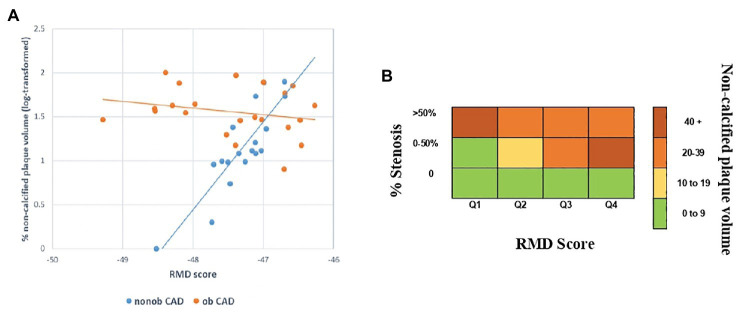
Association between HRP (such as the non-calcified plaque volume) and the RMD score in nonob and ob CAD groups **(A)**. Risk chart, relating the RMD score and the maximal percentage of stenosis with the extent of HRP feature (i.e., the non-calcified plaque volume), showing the existence of a strong relationship in nonob CAD group. The strong association is depicted in dark red, while the weak one in green **(B)**. CAD, coronary artery disease; HRP, high-risk plaque; nonob CAD, non-obstructive coronary artery disease; ob CAD, obstructive coronary artery disease; RMD, RBC morphodynamic; RBC, red blood cell.

The RMD score was not associated with the presence or degree of CAD (Table 3), but it strongly correlated with the extent of HRP (such as the non-calcified plaque volume) in the nonob CAD group (Figure 4A). Therefore, the RMD score could be utilized in clinical practice to illustrate its potential use, we created a risk chart, relating three variables: the RMD score (in quartiles), the max % stenosis, and the non-calcified plaque volume (Figure 4B). This chart shows that only in nonob CAD patients the plaque instability increases according to RMD score quartile. Of note, a significant interaction between the RMD score and patient group was found (value of *p* = 0.001), with an R-square (i.e., the proportion of the variability of non-calcified plaque volume explained by the score) in the nonob CAD group equal to 0.38, whereas in the ob CAD was 0.008.

## Discussion

In this study, we highlighted the presence of changes in morphodynamic features of RBCs from patients with nonob CAD assessed by means of LoRRca. In this clinical setting, RBC alterations correlate well with HRP features detected by CCTA, suggesting the existence of a possible link between erythrocyte morphodynamics and the increased risk of acute coronary events.

In the last decade, a high number of reports highlighted the presence of acute coronary syndrome (ACS) in the contest of nonob CAD. Up to now, the identification of HRPs is demanded to CCTA and, even if preliminary data on the CAPIRE study may suggest a potential association between HRP features and inflammatory markers (Conte et al., 2020a), the lack of circulating biomarkers associated with plaque instability constitutes a critical diagnostic issue.

Our findings, highlighting the existence of a connection between the erythrocyte morphodynamic behavior and plaque instability, turns on new lights on this circulating cell, moving the attention from the instable plaque to the instable patient.

During its long lifespan of 120 days, RBCs pass through the entire circulatory system where they play a key role in blood flow regulation and the consequent tissue perfusion (Yedgar et al., 2002). Even if up to now the precise mechanism underlining the involvement of this blood cell in the control of circulatory processes is not well described, some hypotheses can be formulated. In particular, it has been recognized the role of ATP release from RBC in modulating vasomotor tone in the microcirculation through a diffusive mechanism toward endothelial cells. This mechanical-dependent release is sensitive to several stimuli and, between them, low oxygen level, shear stress, and shape deformation and is believed to play an important role in the microcirculation, characterized by high levels of shear stress and shape deformation degree (Sprague et al., 1998; Dietrich et al., 2000; Wan et al., 2008; Forsyth et al., 2011). Another key mechanism through which RBCs regulate vascular function is NO production and release (Simmonds et al., 2014). Specifically, thanks to the internal compartmentalization of hemoglobin, RBCs are able to maintain hemostasis through the well-regulated delivery of oxygen and the balance of NO scavenging and production (Helms et al., 2018).

It becomes evident the importance of RBC regulatory role in vascular function in pathological states able to compromise the RBC membrane integrity leading to conditions of increased oxidative stress, hypertension, thrombosis, and vaso-occlusion.

In this regard, RBC aggregation and adherence to the endothelial wall were described to be influenced by both blood cellular and plasma factors, as this is a reversible process that depends on the concentration of high molecular weight proteins such as fibrinogen (Baskurt, 2007). In this regard, it has been demonstrated that RBC aggregates strength directly correlates to the inflammatory state (Ami et al., 2001). Specifically, the RBC aggregation parameters considered in the study conducted by Ami and colleagues (i.e., the average aggregate size, the distribution of the RBC population into aggregate size ranges, the shear stress required to obtain 50% of RBC population in the small range of aggregate size, and the area under the curve (AUC) of the plot as a function of shear stress) correlated with the inflammatory indexes C reactive protein and fibrinogen in patients with cardiovascular and infectious conditions associated with an inflammatory response, regardless of the specific pathological state and despite the variability of symptoms. In addition, RBC deformability was found to be able to control platelet-vessel interaction (Aarts et al., 1984). In this context, the morphodynamic characteristics we evidenced in RBCs of patients with nonob CAD could be the result of the interaction among RBC and cell components or humoral factors derived from plaque or are consequent to the alteration of the erythrocyte itself that negatively influences plaque progression (Aarts et al., 1984).

It could be expected HRP features to be associated to RBC dysfunctionality also in ob CAD patients. These data might be explained by the diffuse endothelial dysfunction that characterizes patients with ob CAD that could mask the interplay between atherosclerosis and RBC alterations. Of interest, when comparing individuals with or without ob stenosis, no difference in RBC morphodynamics was observed as total plaque volume was not associated with erythrocyte features, suggesting that overall atherosclerosis burden is a sub-optimal marker of atherosclerosis disease activity at least in the early stages.

Thus, it can be hypothesized that RBC could be mainly involved in the early phase of atherosclerosis process (nonob CAD), rather than in the more advanced stages (ob CAD).

Our data have also to be considered in view of the results of the CAPIRE study that specifically identified high non-calcified plaque volume as the most ACS-predictive parameter in CAD patients. The underlined association between RBC dysfunction indexes and adverse plaque features (i.e., non-calcified plaque volume) can be proposed as a new systemic non-invasive marker for the identification of high risk patients (Andreini et al., 2019).

In this context, one possible pathophysiological model to explain the correlation between elevated non-calcified plaque volume and RBC rigidity could be identified in the role of cholesterol in determining RBC deformability. More precisely, as previously described (Banerjee et al., 1998; Cicco and Pirrelli, 1999; Ercan et al., 2002), higher amount of cholesterol in RBC structure positively correlates with their hemorheological parameters and especially with rigidity. In our clinical setting, the decrease in the area under the curve (AUC) value in ob CAD vs. no CAD could be the result of increased cholesterol (Banerjee et al., 1998), as in our patient group, the levels of LDL cholesterol show a trend to increase, moving from nonob CAD to no CAD and to ob CAD. We cannot exclude that the absence of a difference in the EI max value can be attributable to the pharmacological treatment with statins, present in the 50% of nonob CAD and in the 31.2% of ob CAD.

Similarly, tissue with low CT attenuation are commonly considered as lipid rich and low-attenuation coronary plaque in CCTA could be considered as a diagnostic for lipid-core unstable plaque (Chen et al., 2010; Schlett et al., 2013). This pathophysiological hypothesis, even if of speculative nature, well fits with the widely recognized role of dyslipidemia as a major cardiovascular risk factor.

Another factor able to augment RBC rigidity is an increase in intracellular viscosity because of increased MCHC. Specifically, a variation in MCHC values as a result of a different hydrating states of erythrocytes is able to induce a modification in the osmotic deformability profile (and consequently in the area) without affecting the EImax (Clark et al., 1983). According to these findings, in our study population we registered a significant increase in MCHC values from no CAD to ob CAD that could explain in part this result.

As clinical tool, the morphodynamic characteristics combined into the RMD score could be used in a risk chart. This is a simple, cheap and non-invasive procedure that will allow identifying high risk patients in the early stage of atherosclerosis. The use of this chart in the clinical practice could help in better identifying patients who may merit CCTA, even if asymptomatic, and in the long-term follow-up of non-obstructive patients with a high probability of experiencing acute events.

Several limitations warrant discussion. This study was conducted on a small number of subjects. The RMD score and its relative risk chart here obtained have not to be considered as substitutes of CCTA. Moreover, further studies are needed before our findings could be used in clinical practice for early identification of asymptomatic patients who may merit screening CCTA, that nowadays remains not indicated.

## Conclusion

In nonob CAD patients the HRP features correlated with an altered RBC morphodynamic behavior highlighted by means of LoRRca. Three of the erythrocyte flow-affecting properties analyzed are independent predictors of the extent of the non-calcified plaque volume in this population. The RMD score and the related risk chart clearly highlighted the rise of acute events probability with the increase of erythrocyte altered features.

Further studies are needed to validate this chart in a large cohort of subjects in order to define its potential use in patient identification and management, with the final goal of slowing the disease progression.

## Data Availability Statement

The raw data supporting the conclusions of this article will be made available by the authors, without undue reservation.

## Ethics Statement

The studies involving human participants were reviewed and approved by Local ethics research committee of Centro Cardiologico Monzino. The patients/participants provided their written informed consent to participate in this study.

## Author Contributions

BP, EC, VC, and DA designed the study. BP, AZ, AM, and SF performed the ektacytometry assays. EC and SM enrolled patients. BP, EC, PB, DA, and VC interpreted the results. FV and SB performed statistical analysis. BP and EC wrote the manuscript. BP, EC, AZ, PB, FV, SB, SF, SE, AM, SM, ET, VC, and DA reviewed and approved the final version of the manuscript.

### Conflict of Interest

The authors declare that the research was conducted in the absence of any commercial or financial relationships that could be construed as a potential conflict of interest.
