# Supplementary material for: Red Blood Cell Morphodynamics: A New Potential Marker in High-Risk Patients
Source: Front Physiol. 2021 Jan 13;11:603633. doi: 10.3389/fphys.2020.603633 (PMC7838560; doi:10.3389/fphys.2020.603633)
Supplement: Supplementary file 1 [file Presentation_1.pptx]

## Slide 1
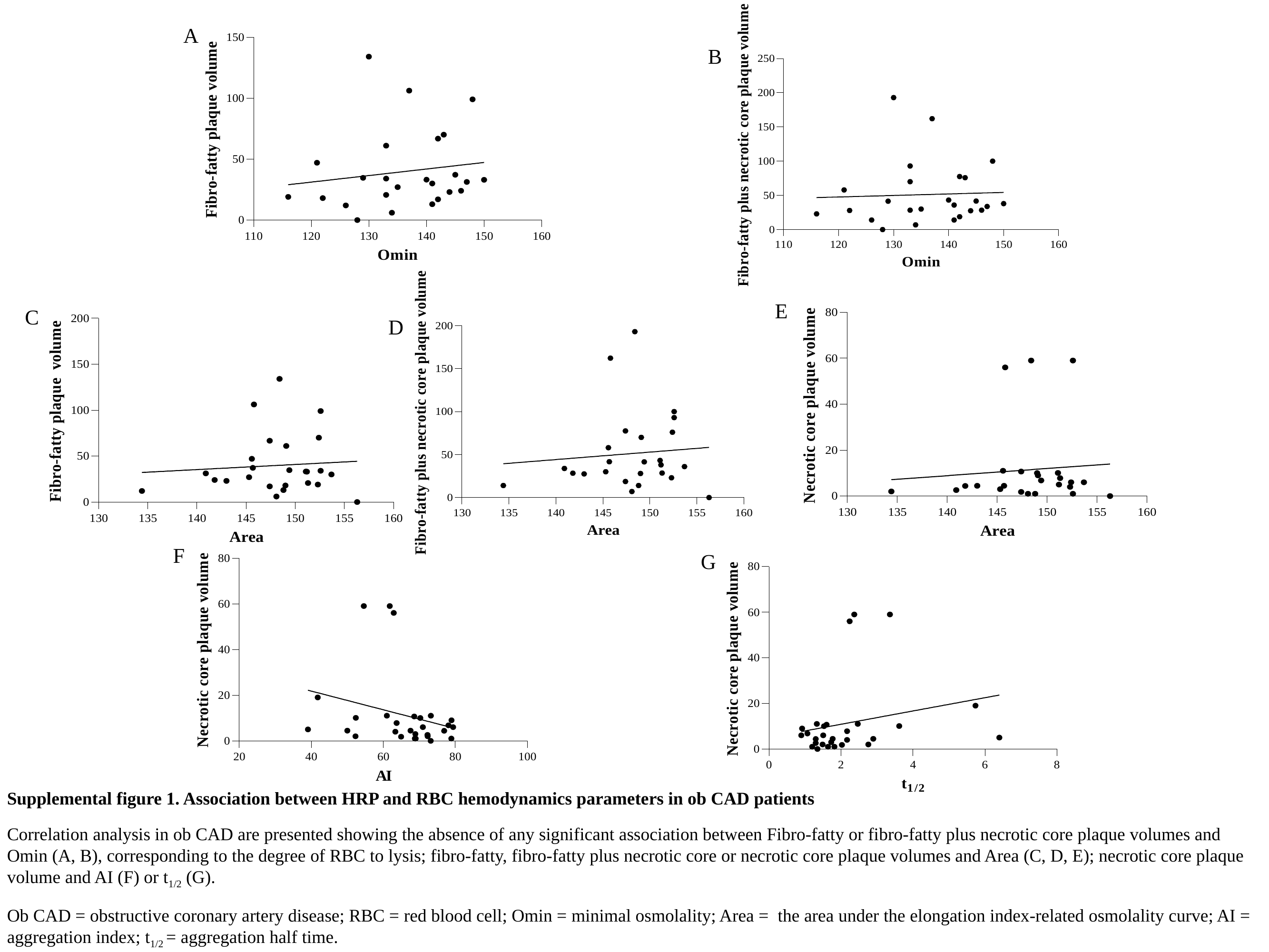

A
B
E
C
D
F
G
Supplemental figure 1. Association between HRP and RBC hemodynamics parameters in ob CAD patients
Correlation analysis in ob CAD are presented showing the absence of any significant association between Fibro-fatty or fibro-fatty plus necrotic core plaque volumes and Omin (A, B), corresponding to the degree of RBC to lysis; fibro-fatty, fibro-fatty plus necrotic core or necrotic core plaque volumes and Area (C, D, E); necrotic core plaque volume and AI (F) or t1/2 (G).
Ob CAD = obstructive coronary artery disease; RBC = red blood cell; Omin = minimal osmolality; Area = the area under the elongation index-related osmolality curve; AI = aggregation index; t1/2 = aggregation half time.

## Slide 2
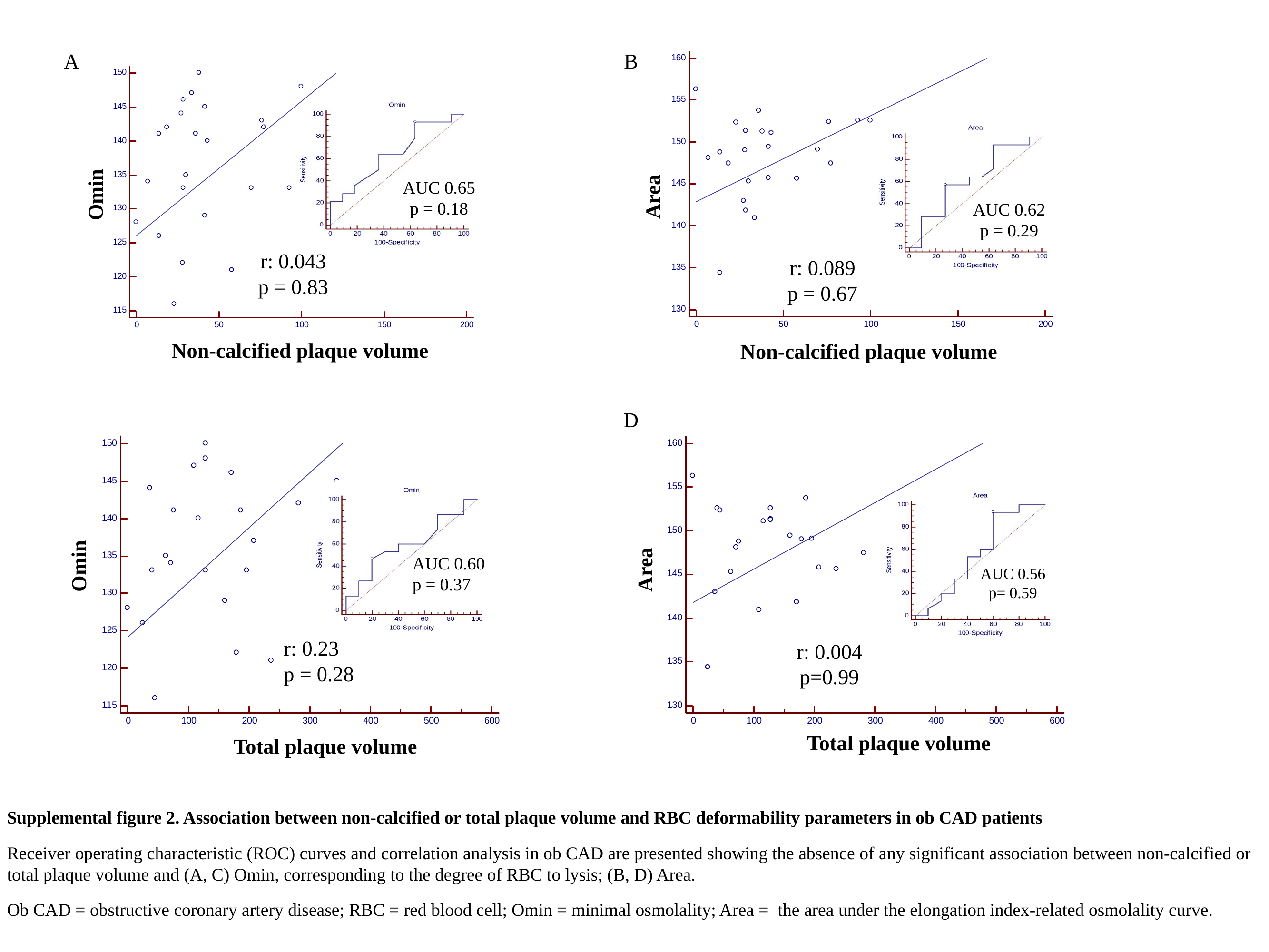

Area
AUC 0.62
p = 0.29
r: 0.089
p = 0.67
Non-calcified plaque volume
AUC 0.65
p = 0.18
Omin
r: 0.043
p = 0.83
Non-calcified plaque volume
A
B
C
D
AUC 0.60
p = 0.37
Omin
r: 0.23
p = 0.28
Total plaque volume
Area
AUC 0.56
p= 0.59
r: 0.004
p=0.99
Total plaque volume
Supplemental figure 2. Association between non-calcified or total plaque volume and RBC deformability parameters in ob CAD patients
Receiver operating characteristic (ROC) curves and correlation analysis in ob CAD are presented showing the absence of any significant association between non-calcified or total plaque volume and (A, C) Omin, corresponding to the degree of RBC to lysis; (B, D) Area.
Ob CAD = obstructive coronary artery disease; RBC = red blood cell; Omin = minimal osmolality; Area = the area under the elongation index-related osmolality curve.
